# Risk Preferences and Predictions about Others: No Association with 2D:4D Ratio

**DOI:** 10.3389/fnbeh.2018.00009

**Published:** 2018-02-01

**Authors:** Katharina Lima de Miranda, Levent Neyse, Ulrich Schmidt

**Affiliations:** ^1^Kiel Institute for the World Economy Kiel, Germany; ^2^SOEP at German Institute for Economic Research (DIW) Berlin, Germany; ^3^Department of Economics and Econometrics, University of Johannesburg Johannesburg, South Africa; ^4^Department of Economics, University of Kiel Kiel, Germany

**Keywords:** risk, decision making, prenatal testosterone, 2D:4D, stereotypes, gender

## Abstract

Prenatal androgen exposure affects the brain development of the fetus which may facilitate certain behaviors and decision patterns in the later life. The ratio between the lengths of second and the fourth fingers (2D:4D) is a negative biomarker of the ratio between prenatal androgen and estrogen exposure and men typically have lower ratios than women. In line with the typical findings suggesting that women are more risk averse than men, several studies have also shown negative relationships between 2D:4D and risk taking although the evidence is not conclusive. Previous studies have also reported that both men and women believe women are more risk averse than men. In the current study, we re-test the relationship between 2D:4D and risk preferences in a German student sample and also investigate whether the 2D:4D ratio is associated with people’s perceptions about others’ risk preferences. Following an incentivized risk elicitation task, we asked all participants their predictions about (i) others’ responses (without sex specification), (ii) men’s responses, and (iii) women’s responses; then measured their 2D:4D ratios. In line with the previous findings, female participants in our sample were more risk averse. While both men and women underestimated other participants’ (non sex-specific) and women’s risky decisions on average, their predictions about men were accurate. We also found evidence for the false consensus effect, as risky choices are positively correlated with predictions about other participants’ risky choices. The 2D:4D ratio was not directly associated either with risk preferences or the predictions of other participants’ choices. An unexpected finding was that women with mid-range levels of 2D:4D estimated significantly larger sex differences in participants’ decisions. This finding needs further testing in future studies.

## Introduction

Human behavior and decision making are closely connected to individuals’ social environment as well as their beliefs about other people’s behaviors, actions, preferences, and characteristics. According to Social Comparison Theory, humans tend to continuously compare themselves with others (Festinger, 1954) and their social identity is connected to these comparisons (see Hogg, 2000). As these comparisons are often made under the influence of erroneous reference points and social stereotypes (Katz and Braly, 1965), inaccurate stereotyping is an inevitable consequence.^1^ Although stereotypes typically affect certain social groups externally, individuals may also influence their own self-concept through self-stereotyping (Latrofa et al., 2010) or stereotype threat (Steele and Aronson, 1995). This means that stereotypes may shape human behavior through diverse social and psychological channels. Alongside numerous types of stereotypes such as ethnic, political or religious, gender has been a significant research topic in various fields of social science, such as psychology, sociology, and economics. Examples include gender stereotypes in management (Powell et al., 2002), social inferences (Berndt and Heller, 1986), negotiation performance (Kray and Thompson, 2004) and risk preference predictions (Siegrist et al., 2002). In the field of economics in particular, gender stereotypes have been the focus of attention as numerous gender gaps are observed in both macroeconomic and microeconomic indices. Typical examples show that the balance is tipped in the favor of men; in income, education, health, political and labor force participation as well as occupied managerial positions as documented in the Global Gender Gap Report 2016 (Leopold et al., 2016).

While gender discrimination plays a major role in gender gaps in economics, there also exists a vast literature pointing out various gender differences in economic behavior. These differences might also have an impact on gender gaps or they may correlate with gender stereotypes, although the extent of causality is vague. One common finding in this regard is the higher risk aversion of women (Byrnes et al., 1999; Croson and Gneezy, 2009; Charness and Gneezy, 2012). According to existing literature, gender stereotypes are attached to gender effects in risk preferences. In Siegrist et al. (2002) for example, participants were asked to estimate other people’s answers in a questionnaire on risk attitudes. Their results show that both men and women overestimated men’s risk preferences; which was a clear sign of being biased by common stereotypes. Ball et al. (2010) also confirmed that the perception of others’ risk attitudes reflected common stereotypes.

That women are found to be more risk averse than men on average has, in recent years, led to curiosity about the biological roots of gender differences in risk attitudes. The role of the steroid hormone testosterone (hereafter T) has been one of the most widely investigated biological foundations. As higher T is associated with more masculine behavior and personality characteristics, the association between T and risk taking has been a common inquiry. Yet, the results are not entirely conclusive due to the complexity of both human endocrinology and decision making processes. The methods used to investigate the relationship between T and financial risk taking are clustered in three categories. First method is to study *circulating T* which has a systematic impact on decision making. However, as it is a continuously fluctuating hormone, the studies focusing on circulating T are mostly limited to correlational findings. *Manipulating the circulating T* is a method of identifying causality. The third method is to study the *organizational role of T* through indirect measurements, such as the 2D:4D ratio of hands. We investigate the association between 2D:4D and risk preferences and also the relationship between 2D:4D and one’s perceptions about other people’s risk preferences. Apicella et al. (2015) reviews the financial risk taking and T literature, while Nadler and Zak (2016) review the role of T in economic behavior in depth.

### Background Literature

#### Stereotyping and Estimating Risk Preferences

While Social Role Theory suggests that the gender differences in behavior and gender stereotypes originate from separate social roles of men and women in society (Eagly and Steffen, 1984; Eagly et al., 2000), a stereotype itself may also drive the target group to confirm that stereotype, even if it is an inaccurate one. This phenomenon, called the *stereotype threat*, may consequently contribute to the persistence of a gender role in society. A common example is mathematical ability. Primed by the gender stereotype suggesting the higher numerical ability of men, female participants perform worse in math tests than their actual potential (Brown and Josephs, 1999; Shih et al., 1999; Spencer et al., 1999). In line with stereotype threat examples in performance, the stereotype suggesting that *men are risk-takers* was also confirmed by women in previous studies.

Siegrist et al. (2002) asked their participants to make sex specific predictions about risk preferences with hypothetical questions. While both men and women made accurate predictions about women’s risk preferences, both overestimated the number of risky choices by men. Interestingly, women’s predictions about the number of risky choices men would make were higher than men’s predictions about their own sex. The seminal study of Eckel and Grossman (2008) experimentally confirmed that both sexes predict male peers would take higher risks than female peers. Although this prediction was accurate, it is an evidence of stereotyping in both sexes. Roszkowski and Grable (2005), Daruvala (2007), and Grossman (2013) support the existence of gender stereotyping in risk attitude predictions in the same direction.

Although the predictions were not sex-specific, the preceding studies investigated predictions about others’ risk preferences. For example, Hsee and Weber (1997) argued that people’s risk preferences are affected by their emotional reactions to risk and that their predictions about others are related to common (cultural) stereotypes. Wallach and Wing (1968) and Levinger and Schneider (1969) showed that people typically believe they are themselves more risk taking than others. This finding was replicated in numerous studies (Clark et al., 1971; Lamm et al., 1972) with the exception of Hsee and Weber (1997) where participants estimated higher risk taking for others than themselves. One explanation for this common finding is the *risk-as-value hypothesis* (Brown, 1965), according to which individuals perceive risk seeking as a culturally more admirable value and therefore their beliefs about themselves and others are biased accordingly. Beliefs about others’ risk preferences also reflect one’s own risk preferences. This effect was termed the *false consensus effect* and is also a commonly observed prediction bias (Ross et al., 1977).

#### 2D:4D Ratio

The fetus’ brain development and endocrine system are influenced by prenatal T exposure and the decision making patterns and personality traits of humans are also partially effected by it (Manning, 2002). Digit ratio (2D:4D) is the ratio between the index and ring fingers and it is employed as an indirect bio-marker of prenatal androgen exposure. A lower 2D:4D ratio indicates a higher level of prenatal T to estradiol ratio (Lutchmaya et al., 2004) and men typically have lower 2D:4D ratios (Hönekopp and Watson, 2010). The negative relationship between prenatal androgen exposure and 2D:4D was confirmed via various methods. For example, Lutchmaya et al. (2004) and Ventura et al. (2013) studied the relationship by taking direct evidence from amniotic fluid samples during pregnancy and linking the endogeneous T and estradiol ratio data to the finger ratios of newborns and infants. Along with previous correlational approaches, the experimental study of Zheng and Cohn (2011) also observed lower 2D:4D ratios in rodents administrated androgen *in utero*. They conclude that sexually dimorphic 2D:4D is caused by androgen and estrogen signaling. In a twin study van Anders et al. (2006) showed that women with male twins have lower 2D:4D than those with female twins. Typically, 2D:4D shows greater sex differences in the right hand (Hönekopp and Watson, 2010). This is why a large majority of the 2D:4D literature is based on samples gathered from right hands. It should also be noted that circulating T and prenatal T do not necessarily correlate. No significant relationship between 2D:4D and adult sex hormones has been observed in the meta-analytical study of Hönekopp et al. (2007).

A number of studies have shown that several typical gender effects in economics were also observed between low and high 2D:4D individuals. Examples include negative relationship between 2D:4D and overconfidence (Dalton and Ghosal, 2014; Neyse et al., 2016), higher success among high-frequency traders (Coates et al., 2009), earnings in economic games (Buser, 2012) and lower degrees of loss aversion (Hermann, 2017). Note that the last two studies, Buser (2012) and Hermann (2017), use self-reported 2D:4D as a measurement method which was criticized in Brañas-Garza and Kovářík (2013).

In the domain of risk preferences, numerous studies also point out negative relationships. Dreber and Hoffman (2007) and Garbarino et al. (2011) show negative associations in both sexes, while Ronay and von Hippel (2010) only for men with incentivized tasks. Brañas-Garza and Rustichini (2011) and Stenstrom et al. (2011) also showed negative relationship for men without incentivized risk elicitation tasks. These results have been confirmed in a recent study with a large sample size and with an incentivized risk elicitation task (Brañas-Garza et al., 2017). However, there are also studies which did not report any significant associations (Apicella et al., 2008; Schipper, 2012; Aycinena et al., 2014; Drichoutis and Nayga, 2015).

One reason behind the conflicting results of these studies can be heterogeneity among (i) risk elicitation methods, (ii) sample sizes and ethnic backgrounds, (iii) incentive mechanisms, and (iv) 2D:4D measurements methods. Above mentioned studies use different risk elicitation tasks such as the Holt and Laury (2005) method Brañas-Garza and Rustichini (2011), Schipper (2012), Aycinena et al. (2014), Drichoutis and Nayga (2015), the Gneezy and Potters (1997) method (Dreber and Hoffman, 2007; Apicella et al., 2008), multiple price lists (Garbarino et al., 2011) or the Balloon Analog Risk Task (Lejuez et al., 2002) method (Ronay and von Hippel, 2010). For example, Filippin and Crosetto (2016) reported that risk elicitation tasks, such as the Holt and Laury method, may fail to detect gender effects. Since 2D:4D is a sexually dimorphic measure, studies using this method may have failed to find a relationship. Furthermore, most of these tasks were employed with real monetary incentives while some (Brañas-Garza and Rustichini, 2011; Stenstrom et al., 2011) were not.

Other possible challenges may be the varying sizes and ethnic backgrounds of the samples. While some of the studies gathered their data from mixed samples, others used Caucasians or non-Caucasians only as the 2D:4D ratio is also reported to be sensitive to ethnic differences (Manning et al., 2004). In addition, using different 2D:4D measurement methods might have had an effect on 2D:4D distributions of the samples. Using scanners, photocopies, calipers, and rulers are the most common methods.

To the best of our knowledge, the relationship between 2D:4D and stereotyping has not been investigated to this date. In the account of circulating T, Josephs et al. (2003) showed that the participants with higher circulating T were more responsive to signals that reminded them of their social status than those with lower T. In their study, participants were primed negatively or positively depending on their sex prior to a math test. Women with higher circulating T who were primed by the low-numerical-ability stereotype performed lower in the math test than their low circulating T peers. Men with higher circulating T on the other hand, performed better when they were primed by high-numerical-ability stereotype than their low T peers. Josephs et al. (2003) suggest that a stereotype is a statement about one’s dominance and status and therefore the effect of circulating T might have been moderated by status concerns. Similar to this finding, Millet and Dewitte (2008) showed that when men with low 2D:4D learn that they are in a subordinate position, they react strongly to excel in their social status. Millet (2009) also highlights that individuals with lower 2D:4D would have a higher need for achievement. Thus, lower 2D:4D may also be associated with a higher level of gender bias about risk preferences.

The current study initially tests the relationship between risk preferences and 2D:4D, using an incentivized Eckel and Grossman risk elicitation method (Eckel and Grossman, 2002). Furthermore, the participants of the study were also asked to make both sex-free and sex-specific predictions about other participants’ choices.

#### Main Hypotheses

When making predictions about other people’s preferences, individuals typically base their predictions on their own preferences and on stereotypes. In this regard, several studies have found that people typically believe that they are themselves more risk taking than others (Wallach and Wing, 1968; Levinger and Schneider, 1969; Clark et al., 1971; Lamm et al., 1972), resulting in the finding that the predictions of other people’s risk taking is lower than own risk taking. One explanation for this common finding is the risk-as-value hypothesis (Brown, 1965), according to which, individuals perceive risk taking as a cultural value and therefore their beliefs about themselves and others are also biased accordingly.

*Hypothesis 1: Participants take higher risk than they estimate others to take.*

Another commonly observed phenomenon is that people rely on their own risk preferences when making predictions about others. This implies a positive relationship between risk preferences and the predictions about other people’s risk preferences (false consensus effect, e.g., Ross et al., 1977).

*Hypothesis 2: Participants’ risk preferences correlate positively with their estimations about others.*

In keeping with the wealth of such findings in the literature (Byrnes et al., 1999; Croson and Gneezy, 2009; Charness and Gneezy, 2012) we expect to observe higher levels of risk aversion in women. Although Filippin and Crosetto (2016) report that the magnitude and importance of this gender effect is debatable and seems to be task-specific, the task employed in this study has resulted in consistent gender differences in earlier studies.

*Hypothesis 3: Men’s choices are less risk averse than women’s.*

Considering the previously discussed inconclusive results on the association between 2D:4D and risk preferences we re-examine whether lower 2D:4D ratios are associated with higher risk taking.

*Hypothesis 4: 2D:4D is negatively correlated with risk taking.*

While the relationship between risk taking and 2D:4D has been tested in a number of studies, the relationship between 2D:4D and the perception of other people’s risk preferences has not been examined so far, to the best of our knowledge. To predict other people’s preferences, individuals often rely on their personal preferences as well as stereotypes. Stereotypically women should be risk averse and the opposite holds for men. Following the earlier discussion, we examine if participants with lower 2D:4D react more strongly to sex information than people with high 2D:4D ratios and, therefore, over-estimate women’s risk aversion as well as men’s risk taking.

*Hypothesis 5: The difference between predictions about men and women is negatively correlated with 2D:4D.*

## Materials and Methods

### Participants and Procedures

The experiment was carried out in June 2017 at the Experimental Lab of Kiel University. 150 students from Kiel University participated in a total of 10 sessions and each participant participated only in one session of the experiment. Given the mixed evidence on the relation between 2D:4D and risk taking, the sample size was chosen in order to assure sufficient power to determine a relatively small effect size. Our correlation power analysis suggested a minimum sample size of 125 (α = 0.05 – type I error, β = 0.20 – type II error, *r* = 0.25). Participants were recruited from the subject pool of the Experimental Lab Kiel with the software package hroot (Bock et al., 2014). Students from different faculties took part in the experiment with the majority (37%) studying economics, followed by students from the philosophy faculty (27%) and STEM fields (21%). The experiment as such was paper based and each session lasted approximately 30 min and had on average 17 participants (minimum 12 and maximum 20 participants per session). Participants received a show-up fee of €3.00 and could additionally win up to €13.00 depending on their responses. Gender distribution was almost balanced with 72 participants who indicated they were male and 74 female, while four participants did not specify their sex. Average age was 26 years (*SD* = 3.17 and 95% confidence interval [25.30; 26.33]).

All participants of the experiment were informed with a written form about the content and the protocol of the study before participation. Participation and the hand scanning were completely voluntary and the participants were free to leave the experiment with their participation fee any time they wanted. Opting out from the hand scanning did not affect participants’ pay. Anonymity was preserved by assigning the participants a randomly generated code that cannot be associated with any personal information or decision, either in the experiment or in the hand scanning. An ethical review and approval was not required for this study in accordance with the local legislation and institutional guidelines. As is standard in economics experiments, no ethical concerns were involved other than preserving the anonymity of the participants. Each participant signed a receipt of his/her payment at the end of the experiment. The whole protocol was performed in accordance with the ethical guidelines of the Kiel University Experimental Economics Lab, where it was approved by the lab manager.

#### Risk Preferences and Predictions

To elicit risk preferences, the method developed by Eckel and Grossman (2002) was used (hereafter EG). Participants were confronted with six lotteries and had to choose one of them (**Table 1**). Each lottery had a 50% chance to win and a 50% chance to loose. The expected value of the lotteries increased from lottery 1 to 5 as well as the variance, lottery 6 had the same expected value as lottery 5 but a higher variance.^2^ The higher the EG choice, the lower is the degree of risk aversion (reflected by the increase in variance from lottery 1 to 6). The participants were informed that their decision would be pay-out relevant, as at the end of the experiment a coin would be thrown and depending on the result the higher or lower amount would be paid out.

**Table 1:** EG risk elicitation task.

Lotteries (50/50 chance)	Low payoff	High payoff	Expected value
Lottery 1	€ 4.00	€ 4.00	€ 4.00
Lottery 2	€ 3.50	€ 5.00	€ 4.25
Lottery 3	€ 3.00	€ 6.00	€ 4.50
Lottery 4	€ 2.50	€ 7.00	€ 4.75
Lottery 5	€ 2.00	€ 8.00	€ 5.00
Lottery 6	€ 1.00	€ 9.00	€ 5.00

After this incentivized risk elicitation, participants were asked to estimate which lottery was chosen on average by other participants, which lottery men chose on average and which lottery women chose on average. In addition, the participants filled out a short questionnaire about general demographic information, life satisfaction, mindfulness, social comparison, and cooperation. At the end of the protocol participants were anonymously paid and their hands were scanned for 2D:4D measurement.

#### 2D:4D Ratio

At the end of the protocol, both hands of each participant were scanned with a flatbed scanner. All participants were individually briefed about the scanning procedure and 2D:4D literature prior to the scans. The scanning was voluntary and one participant chose to opt out from the hand-scan. We followed Neyse and Brañas-Garza (2014) scanning and measuring protocol precisely. The scans were measured two times in GIMP software blindly (by generated participation numbers) and in a random order by a trained research assistant. There were 2 weeks between the first and the second measurements and we ensured that the measurements were recorded on blank paper to avoid framing effects and post-measure corrections. Both measurements were highly correlated (>0.95). The mean of the two measures was taken as the main 2D:4D variable.

The average right hand 2D:4D is 0.964 (*SD* = 0.031). Men have an average 2D:4D of 0.957 (*SD* = 0.030) and women of 0.971 (*SD* = 0.032). A classic *t*-test rejects equality (*p* = 0.012, *t_143_* = -2.553; *d* = -0.424). The left hand 2D:4D is 0.964 (*SD* = 0.039). Men’s average left 2D:4D is 0.960 (*SD* = 0.029) and women’s is 0.966 (*SD* = 0.046). The difference is lower for the left hand but in the typical direction (*p* = 0.379, *t_142_* = -0.8821; *d* = -0.147). As men usually have lower 2D:4D ratios than women, these differences are in line with the previous literature (see Hönekopp and Watson, 2010 for a meta-analysis of sex differences in 2D:4D). The meta-analysis of Hönekopp and Watson (2010) also concludes that 2D:4D shows a greater difference on the right hand. This is why a big majority of the previous studies based their analysis on right hand measures. Although our main analysis is also based on the right hand, we also report the identical analysis for the left hand in tables and in the Appendix.

As ethnicity plays an important role in 2D:4D (Manning et al., 2004), many studies base their analysis on single-ethnicities. The follow-up questionnaire included an item where participants were asked to indicate their ethnicities. According to the results 134 reported themselves as Caucasian (90.54%), 7 mixed (4.73%), and 3 Asian (2.03%). The remaining participants either did not fill in the item or belonged to different ethnicities. As our robustness checks with only Caucasian participants did not significantly differ from the results with the whole sample, the reported analysis includes the whole sample without any ethnicity restrictions. The statistical analysis of 2D:4D is based on 145 participants as 1 participant had a hand injury and another 4 did not fill in the sex item in the questionnaire. Among the latter, one participants opted out from the hand-scan.

## Results

We will first present our correlation analysis of risk preferences and predictions. Further, we will compare the choices of men and women with *t*-tests. The relationships between 2D:4D and participants’ choices will be investigated both with correlation and regression analyses. Finally, we will test the association between participants’ 2D:4D and their predictions about sex differences in the task with both correlation and regression analyses. In line with the majority of previous studies, our analyses will be based on right hand ratios. However, we will also present the same analysis for the left hand in tables and the Appendix. Complete distributions of the variables can also be found in the Appendix.

### Descriptive Analysis of Risk Taking and Predictions

**Table 2** presents the descriptive statistics of the main variables for all participants in the study. The participants on average chose 3.080 in the six item Eckel and Grossman task. Their predictions about other participants were on average 2.160. The difference between the two variables is significant (*t_149_* = 6.132; *p* < 0.001; *d* = 0.598). This supports *Hypothesis 1* which postulated that participants take higher risk than they estimate others to take. Pairwise correlations show a significant positive correlation between participants’ own choices and their predictions about others (*r* = 0.304, *p* < 0.01). This result supports *Hypothesis 2*. The average prediction about men was 3.873 and about women it was 1.740. Sex-specific predictions correlate both with EG choices (*p* < 0.01 for both) and sex-free predictions (*p* < 0.01 for both).

**Table 2:** Descriptive statistics.

					Pairwise correlation coefficients					
	Variable	Obs	Mean	*SD*	1	2	3	4	5	6
1	Risk	150	3.080	1.774	1.000					
2	Prediction others	150	2.160	1.259	0.304*	1.000				
3	Prediction men	150	3.873	1.420	0.222*	0.282*	1.000			
4	Prediction women	150	1.740	1.089	0.330*	0.696*	0.187	1.000		
5	Right 2D:4D	148	0.964	0.031	-0.102	-0.021	-0.063	-0.019	1.000	
6	Left 2D:4D	148	0.964	0.039	-0.066	0.013	-0.118	0.066	0.502*	1.000

*^∗^p < 0.01.*

### Descriptive Analysis of Risk Taking and Predictions by Sex

**FIGURE 1:**
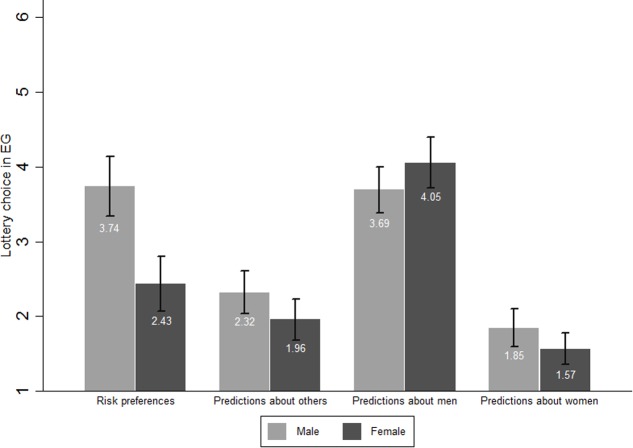
Mean values of choices in EG Task and predictions by sex. Means of risk variables and their 95% confidence intervals grouped by sex.

**Tables 3,3A** and **3B** present the descriptive statistics for men and women separately while **Figure 1** shows the mean values of choices in the EG Task and predictions by sex. In the EG task, men chose 3.736 on average and women’s mean choice was 2.432. This difference, suggesting that women are more risk averse than men, is statistically significant (*p* < 0.001). This finding confirms *Hypothesis 3*.

**Table 3:** Descriptive statistics by sex.

	Men			*t*-tests	Women		
Variable	Obs	Mean	*SD*	Between men and women	Obs	Mean	*SD*
Risk	72	3.736	1.728	*p* < 0.001, *t_144_* = 4.736; *d* = 0.784	74	2.432	1.597
Prediction others	72	2.319	1.243	*p* = 0.039, *t_144_* = 1.782; *d* = 0.077	74	1.959	1.199
Prediction men	72	3.694	1.328	*p* = 0.1248, *t_144_* = -1.544, *d* = -0.256	74	4.054	1.479
Prediction women	72	1.847	1.109	*p* = 0.1, *t_144_* = 1.658, *d* = 0.275	74	1.568	0.923
Right 2D:4D	72	0.958	0.029	*p* = 0.012, *t_143_* = 2.553*; d* = -0.424	73	0.971	0.032
Left 2D:4D	71	0.960	0.028	*p* = 0.379, *t_142_* = -0.882; *d* = -0.147	73	0.966	0.046

					**Pairwise correlation coefficients**					
	**Variable**	**Obs**	**Mean**	***SD***	**1**	**2**	**3**	**4**	**5**	**6**

**(A)** **Descriptive statistics men**

1	Risk	71	3.746	1.738	1.000					
2	Prediction others	71	2.324	1.251	0.315*	1.000				
3	Prediction men	71	3.704	1.335	0.382^∗^	0.427^∗^	1.000			
4	Prediction women	71	1.845	1.117	0.309^∗^	0.700^∗^	0.389^∗^	1.000		
5	Right 2D:4D	71	0.957	0.030	-0.068	-0.056	-0.001	-0.138	1.000	
6	Left 2D:4D	71	0.960	0.029	-0.140	0.061	-0.153	0.021	0.687^∗^	1.000

**(B)** **Descriptive statistics women**

1	Risk	73	2.425	1.607	1.000					
2	Prediction others	73	1.959	1.207	0.181	1.000				
3	Prediction men	73	4.068	1.484	0.170	0.164	1.000			
4	Prediction women	73	1.562	0.928	0.259	0.628^∗^	-0.033	1.000		
5	Right 2D:4D	73	0.971	0.032	0.001	0.092	-0.192	0.172	1.000	
6	Left 2D:4D	73	0.966	0.046	0.012	0.004	-0.137	0.100	0.389^∗^	1.000

*^∗^*p* < 0.01*.

Men’s mean predictions about other participants (2.319) was slightly higher than women’s mean predictions (1.959; *p* = 0.039). On the one hand, men’s average prediction for other men was 3.694 and women’s average prediction for men was 4.054. The equality between men’s and women’s predictions for men cannot be rejected (*p* = 0.125). On the other hand, men’s average prediction for women’s choices was 1.847 and women’s average prediction for other women was 1.568. The equality between the two cannot be rejected (*p* = 0.1).

The equality between men’s actual choices and their predictions about men’s risk preferences cannot be rejected either (*p* = 0.839). This result is also valid for women’s predictions for men (*p* = 0.234). However men’s predictions for women were significantly lower than women’s actual choices (*p* = 0.011) and the same holds for women (*p* < 0.001).

### Analysis of 2D:4D and Risk Preferences

In *Hypothesis 4*, we proposed a negative correlation between the two variables concerning the relationship between risk taking and 2D:4D. Our correlation analysis presented in **Table 2** failed to detect any significant relationship between right (left) 2D:4D and risk (*r* = -0.102, *p* = 0.215 and *r* = -0.066, *p* = 0.429). Furthermore, we did not observe any significant linear relationship between 2D:4D and our three prediction variables in either of the sexes. Therefore, *Hypothesis 4* is rejected.

To further assess the relationship between 2D:4D and risk taking we ran a series of regression models (see Supplementary Table A1). To test the non-monotonic associations we included the quadratic form of 2D:4D in the regression analysis and controlled our models for gender effects. The results remained insignificant for both hands and also for 2D:4D-squared (*p* > 0.1 for all 2D:4D variables).

### Correlation Analysis of 2D:4D and Gender Biases

We relate 2D:4D to the difference between predictions about men and women. To do so, we generated a *gender bias* variable by subtracting predictions about women from predictions about men. Looking at the raw correlations we observe a slight but insignificant correlation between right (left) 2D:4D and the difference in predictions about men and women (*r* = -0.042, *p* = 0.609 and *r* = -0.148, *p* = 0.074) and therefore we reject *Hypothesis 5* which postulated a negative correlation between 2D:4D and gender biases.

### Regression Analysis of Predictions and 2D:4D

Following our correlation analyses, we also ran an additional exploratory OLS regression analysis to investigate non-monotonic associations between predictions that participants made about other people’s risk preferences and their right hand 2D:4D ratios. The dependent variable is sex-free predictions in the first four models. The latter four models investigate the association between participants’ 2D:4D ratios and their predictions about the risk preference difference between the two sexes. The dependent variable is *gender bias*. First independent variable is *risk* which captures the risk preference of each participant measured by choices in the EG task. Second independent variable is 2D:4D and the third is the square of 2D:4D to observe non-monotonic relationship between 2D:4D and dependent variables. Sexes of the participants are controlled for with the dummy variable *female*. The interaction variable *2D:4Dxfemale* is also included in the models to disentangle the impact of sex on the findings about 2D:4D.

The results are shown in **Table 4**. In Models 1–4 we look at the relationship between predictions about other people’s risk preferences without specifying sex. Neither 2D:4D, nor 2D:4D-squared are significant in the first four models (*p* > 0.1 in all of them). Therefore, we may conclude that no monotonic or non-monotonic association between 2D:4D and sex-free predictions is observed. The *female* variable is also not statistically significant in any of these models. The positive and significant coefficients for personal risk taking show that participants base their predictions about others on their personal preferences (*p* < 0.01 in all four models).

**Table 4:** Regression analysis of right hand 2D:4D, risk predictions and gender bias in predictions.

Model Dependent variable	(1) predictions	(2) predictions	(3) predictions	(4) predictions	(5) gender bias	(6) gender bias	(7) gender bias	(8) gender bias
Risk	0.185	0.183	0.185	0.183	0.053	0.060	0.047	0.053
	(0.005)	(0.006)	(0.005)	(0.006)	(0.585)	(0.521)	(0.611)	(0.559)
2D:4D	1.137	-1.621	-3.334	26.189	-5.105	5.368	490.956	415.175
	(0.715)	(0.699)	(0.980)	(0.847)	(0.297)	(0.235)	(0.001)	(0.012)
2D:4D^2^	-	-	2.306	-14.444	-	-	-255.847	-212.852
	-	-	(0.973)	(0.837)	-	-	(0.001)	(0.013)
Female	-0.133	-5.073	-0.132	-5.425	0.795	19.559	0.784	14.369
	(0.512)	(0.404)	(0.514)	(0.377)	(0.004)	(0.023)	(0.004)	(0.092)
2D:4D X female	-	5.124	-	5.489	-	-19.464	-	-14.090
	-	(0.417)	-	(0.388)	-	(0.029)	-	(0.112)
Constant	0.539	3.186	2.704	-10.185	6.537	-3.516	-233.639	-200.554
	(0.856)	(0.430)	(0.966)	(0.877)	(0.174)	(0.424)	(0.002)	(0.011)
R squared	0.084	0.088	0.084	0.088	0.055	0.089	0.103	0.119
*N*	145	145	145	145	145	145	145	145

*OLS regressions, dependent variables are predictions about others [1,6] in the first four models and sex differences in predictions (gender bias = predictions about men – predictions about women). 2D:4D^*2*^ is the square of 2D:4D for quadratic models and 2D:4D X female is the interaction variable for 2D:4D and female. p-Values are given in parentheses*.

**FIGURE 2:**
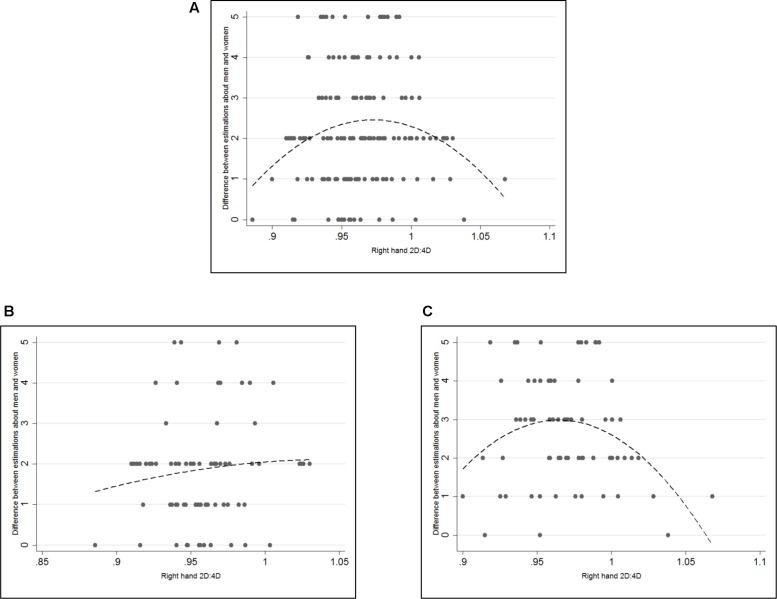
Scatterplots of 2D:4D and *gender bias* in predictions. **(A)** Gender bias in predictions: difference between predictions for men and predictions for women. **(B)** Men’s gender bias in predictions. **(C)** Women’s gender bias in predictions. Dashed lines represent the fitted quadratic models without control variables. Both *2D:4D* and 2D:4D-squared are significant at 99% significance level in **(A)** and at 95% significance level in **(C)**. Neither variable is significant in **(B)**.

This is further assessed in **Table 4** for Models 5–8. The significant coefficients for female participants show that female participants tend to predict a higher difference between men’s and women’s risk taking than male participants (*p* < 0.005 in Models 5 and 7 and *p* = 0.023 in Model 6). As for raw correlations we do not observe a significant coefficient for *2D:4D* in Models 5 and 6 (*p*-values are 0.297 and 0.235 respectively). Models 7 and 8, however, show that there seems to be an inverted U-shaped relationship between 2D:4D and sex difference in predictions. 2D:4D has significant and positive coefficients in both models (*p*-values = 0.001 and 0.012 respectively). 2D:4D-squared on the other hand has significant, negative coefficients (*p*-values = 0.001 and 0.013 respectively). In **Figures 2A–C** scatter plots are shown with the difference between predictions about men and women on the y-axis and right hand 2D:4D on the x-axis.^3^ The dashed lines represent fitted quadratic models. It becomes clear that the quadratic relationship is driven by female participants where low and high 2D:4D women seem to predict a smaller difference in risk taking than women with mid-range 2D:4D ratios. The complete regression analysis on sex specific predictions can be found in Supplementary Table A2 and regressions with left hand measures in Supplementary Table A3.

## Discussion

The main objective of this study was to shed light on the relationship between 2D:4D, risk taking and also predictions about risk taking of other individuals. We initially tested three common findings in the risk literature and found support for all three: (i) The (sex-free) predictions about other participants’ choices were significantly lower than own choices (Wallach and Wing, 1968; Levinger and Schneider, 1969; Clark et al., 1971; Lamm et al., 1972), (ii) participants’ predictions positively correlated with their own choices, which is a finding in support of the false-consensus effect (Krueger and Clement, 1994), (iii) men’s choices were more risk seeking than women (Byrnes et al., 1999; Croson and Gneezy, 2009; Charness and Gneezy, 2012). These findings support our first three hypotheses.

The participants also stated their predictions about men’s and women’s choices in the task. The results show that both men and women estimated the choices of men correctly whereas the predictions about women were significantly lower than women’s actual choices. Underestimation of women’s risk taking behavior is commonly observed in the existing literature (Roszkowski and Grable, 2005; Daruvala, 2007; Eckel and Grossman, 2008; Grossman, 2013).

We then re-tested the connection between participants’ 2D:4D and their own risk taking. No significant relationship between 2D:4D and risk taking were observed in the current study as in Apicella et al. (2008), Schipper (2012), Aycinena et al. (2014), and Drichoutis and Nayga (2015). Due to this result we reject our *Hypothesis 4*. We did not observe a significant relationship between 2D:4D and sex-free predictions either.

As gender biases may be connected to one’s perceptions about others, a possible relationship between 2D:4D and biased predictions were also tested. While 2D:4D did not correlate with predictions, we also ran the same analysis with quadratic models to investigate possible non-monotonic associations between 2D:4D and sex-free predictions. Yet, no non-monotonic association was observed either. However, our *gender bias* variable showed significant, non-monotonic results for women. The inverted U-Shape pattern suggests that female participants with mid-range 2D:4D ratios estimated a higher difference between men and women’s risk preferences than those with high or low 2D:4D ratios. This unanticipated non-monotonic result calls for further investigation as the relationship between 2D:4D and beliefs about other people’s risk preferences has not been investigated before.

There are several studies on 2D:4D that showed non-monotonic results in various contexts. Brañas-Garza et al. (2013) observed an inverted U-Shape pattern between altruism and 2D:4D in both sexes, where the results were more consistent for men than women. This pattern showed that the participants with low and high values of 2D:4D decided to give less money in the dictator game than those with mid-range values of 2D:4D. The same inverted U-Shape pattern between altruism and 2D:4D was also confirmed for both sexes with a larger and multi-ethnic sample in Galizzi and Nieboer (2015). Moreover, in Sanchez-Pages and Turiegano (2010) the individuals with mid-range levels of 2D:4D cooperated more often in the Prisoner’s Dilemma Game. Nye et al. (2012) also showed non-linear associations between 2D:4D and academic performances in samples from Manila and Moscow. In the account for circulating T, Stanton et al. (2011) showed that individuals with low or high levels of circulating T were risk and ambiguity neutral, whereas those with mid-range levels of T were more risk and ambiguity averse. Sapienza et al. (2009) also discussed non-linear associations between risk preferences and circulating T. Furthermore, non-linear associations between salivary T concentrations and visuospatial performance were found in Moffat and Hampson (1996), and between salivary T concentrations and cardiovascular health in Laughlin et al. (2010).

One possible explanation behind non-monotonic relationships between 2D:4D and certain types of behavior may be evolutionary optimization (Alexander, 1996; Sutherland, 2005). Laughlin et al. (2010) discusses the mechanisms behind the non-linear effects of T through the relationship between androgen receptor density and neurotransmitter receptor GABA-A, which has been associated with decision patterns in humans (Lane and Gowin, 2009). As Manning et al. (2003) have shown associations between 2D:4D and androgen receptor gene, the androgen receptor density argument may also be an alternative explanation for non-linearities in 2D:4D studies. McFadden (2002) discusses the non-monotonic impacts of androgen exposure on both humans and animals in detail.

While our results support the conventional findings in the economics literature, we did not find any clear relationship between 2D:4D and risk preferences. The novelty of the current study was its inclusion of perceptions about other people’s risk preferences in the analysis and controlling for sex-specific predictions. We did not find any significant linear relationship between 2D:4D and any of the prediction variables. An unanticipated finding was the inverted U-shaped pattern between 2D:4D and our generated *gender bias* variable for only women in the sample. According to this result women with low or high levels of 2D:4D predicted a smaller difference between men and women’s risk preferences than women with mid-range levels of 2D:4D. Although this relationship has not been investigated before in the literature, it may initiate a new discussion on the link between 2D:4D and decision making under the impact of stereotypes.

As discussed earlier, studies examining the relationship between 2D:4D and risk preferences lack methodological consistency. Several studies use self-reported risk elicitation methods, while some others employ incentivized risk elicitation tasks. Neyse et al. (2016) and Brañas-Garza et al. (2017) showed that the behavior effected by 2D:4D is highly sensitive to monetary incentives. Thus, altering incentives may be one of the reasons behind the lack of consensus. While analyzing decision making under risk, Prospect Theory and Cumulative Prospect Theory (Tversky and Kahneman, 1975, 1992) take into account reference dependence, rank dependence and sign dependence; as risk-taking is closely connected with several other concepts such as loss aversion, ambiguity aversion, or non-linearity in utility. However, risk elicitation tasks used in previous studies have been unable to identify the association between 2D:4D and risky decisions. This is also one of the shortcomings of the current study.

Our results contribute to the growing literature on the biological underpinnings of economic behavior. Since the association between 2D:4D and risk preferences is still not clear, more detailed and systematic investigation on the connection between T and decision making under risk is needed. In this regard, we provide evidence on the gender biased predictions about others’ risk taking. Several studies have pointed out that social comparisons shape risk preferences (Hill and Buss, 2010) and knowledge of income inequality has a higher impact on risk taking than the income itself (Schmidt et al., 2015). In keeping with this evidence, social underpinnings of risk preferences may also be associated with 2D:4D. As stereotypes shape economic life and decisions (see for example Fershtman and Gneezy, 2001; Andreoni and Petrie, 2008) studying the biological roots of stereotyping could also help explain important economic phenomena.

Another limitation of our study is the representativeness bias in student samples. Although a majority of experimental studies are conducted with university students, the representativeness problem is still considered a major drawback in economics experiments. See Levitt and List (2007) for a detailed discussion on laboratory experiments and also Exadaktylos et al. (2013) for a representativeness analysis of self-selected student samples. Although, the findings in 2D:4D literature give important insights into the biological factors of human behavior, the results are both context and sample dependent. Therefore, one should be careful about drawing general conclusions from these findings. Last but not least, the majority of the studies in the literature suffer from small sample sizes and lack of ethnic diversity; limitations which also apply to the current study.

## Author Contributions

All authors listed have made a substantial, direct and intellectual contribution to the work, and approved it for publication.

## Conflict of Interest Statement

The authors declare that the research was conducted in the absence of any commercial or financial relationships that could be construed as a potential conflict of interest.

## Footnotes

1 Even though they are often inaccurate, stereotypes may serve as facilitators in social cognition similar to heuristics and biases in decision making (Tversky and Kahneman, 1975). Judd and Park (1993) provide a thorough discussion of the definition and accuracy of stereotypes.

2 The participants’ choice of lottery number will be referred to as the “EG choice.”

3 For better representation, three observations with negative differences between prediction about men and women’s risk taking were omitted. These observations are however included in the regression analysis in **Table 4** and the inclusion or omission of the observations makes no difference to the qualitative results.
