# Supplementary material for: Risk Preferences and Predictions about Others: No Association with 2D:4D Ratio
Source: Front Behav Neurosci. 2018 Feb 1;12:9. doi: 10.3389/fnbeh.2018.00009 (PMC5810266; doi:10.3389/fnbeh.2018.00009)
Supplement: Supplementary file 1 [file Data_Sheet_1.docx]

**Appendix**

**Figure A1:** Distributions of choices in EG task

| Distribution of choices | Distribution of choices by gender |
| --- | --- |
| 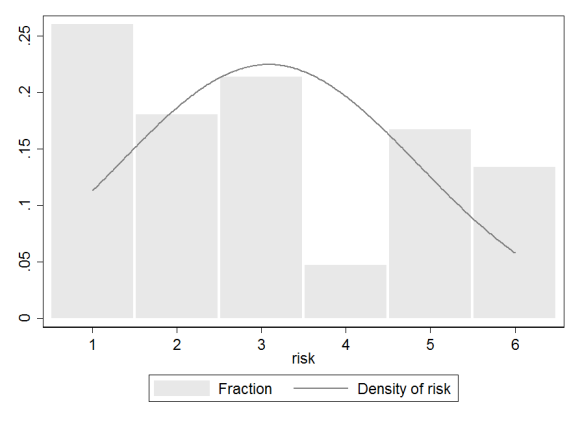 | 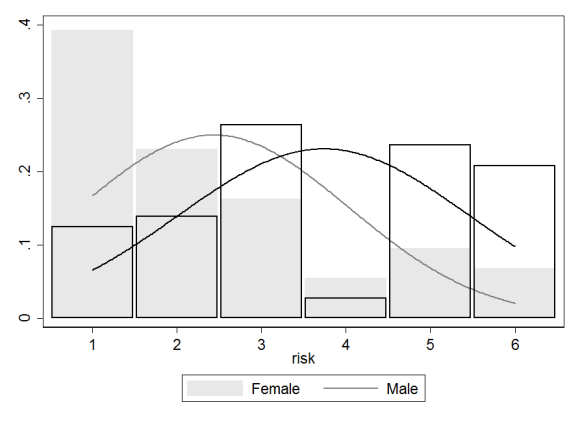 |

**Figure A2:** Distributions of gender-free predictions about others

| Distribution of predictions about others | Distribution of predictions of others by gender |
| --- | --- |
| 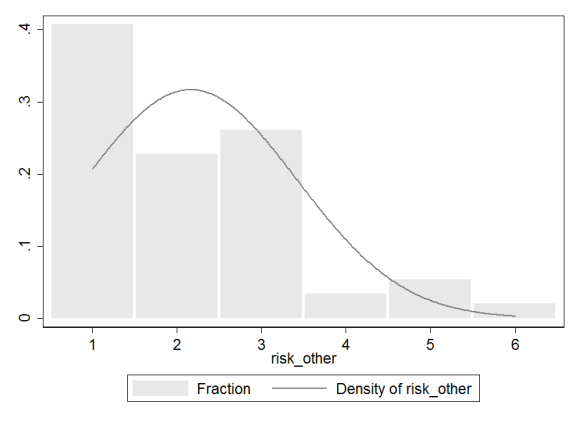 | 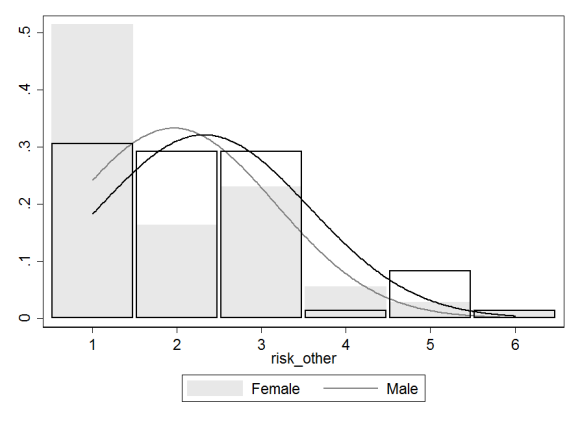 |

**Figure A3:** Distributions of predictions about men

| Distribution of predictions about men | Distribution of predictions of men by gender |
| --- | --- |
| 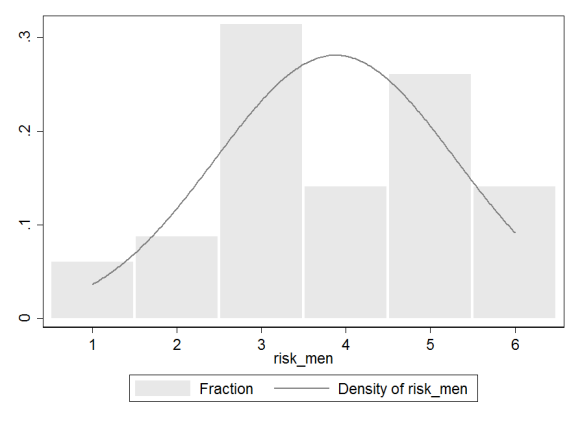 | 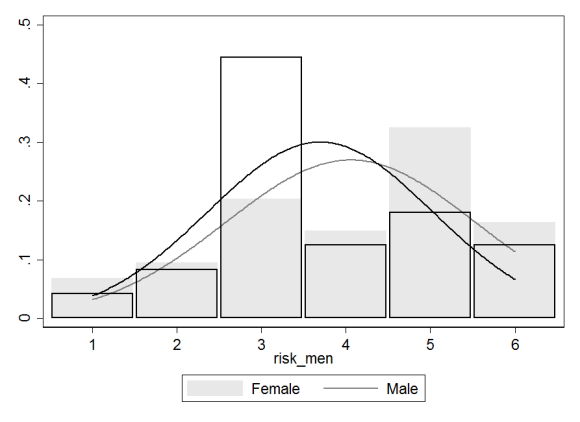 |

**Figure A4:** Distributions of predictions about women

| Distribution of predictions about women | Distribution of predictions of women by gender |
| --- | --- |
| 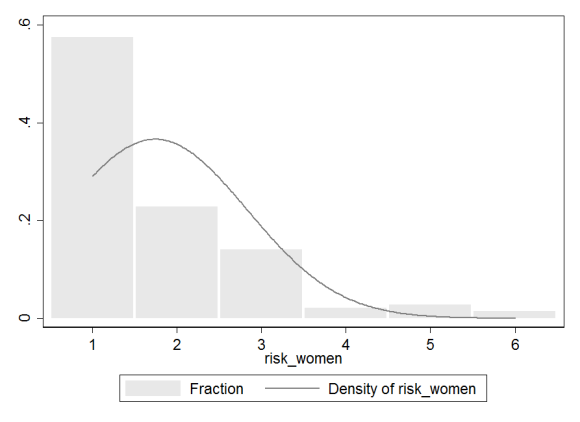 | 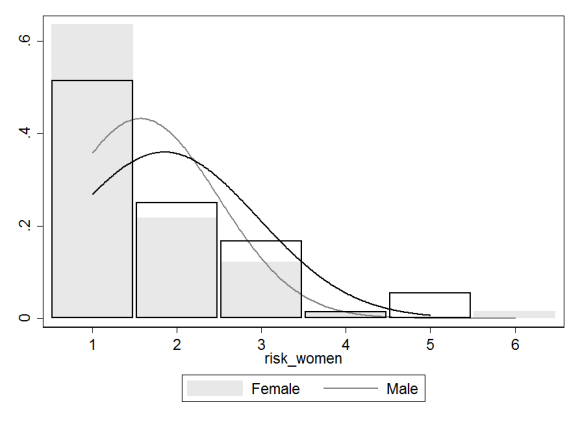 |

**Table A1:** Regression analysis of 2D:4D and risk taking

| Model |  | (1) | (2) | (3) | (4) |  | (5) | (6) | (7) | (8) |
| --- | --- | --- | --- | --- | --- | --- | --- | --- | --- | --- |
| Dependent variable:  *Risk* |  | Right hand 2D:4D | | | |  | Left hand 2D:4D | | | |
| *2D:4D* |  | *-1.816* | *-3.959* | *63.116* | *91.103* |  | *-2.013* | *-8.568* | *13.287* | *-20.764* |
|  |  | *(0.688)* | *(0.533)* | *(0.724)* | *(0.602)* |  | *(0.469)* | *(0.173)* | *(0.644)* | *(0.632)* |
| *2D:4D^2^* |  | *--* | *--* | *-33.489* | *-49.372* |  | *--* | *--* | *-8.395* | *6.323* |
|  |  | *--* | *--* | *(0.718)* | *(0.586)* |  | *--* | *--* | *(0.621)* | *(0.774)* |
| *female* |  | *-1.288* | *-5.131* | *-1.288* | *-6.327* |  | *-1.310* | *-9.937* | *-1.294* | *-10.834* |
|  |  | *(0.000)* | *(0.551)* | *(0.000)* | *(0.450)* |  | *(0.000)* | *(0.140)* | *(0.000)* | *(0.196)* |
| *2D:4D X female* |  | *--* | *3.988* | *--* | *5.228* |  | *--* | *8.969* | *--* | *9.888* |
|  |  | *--* | *(0.657)* | *--* | *(0.549)* |  | *--* | *(0.200)* | *--* | *(0.256)* |
| *constant* |  | *5.475* | *7.527* | *-25.966* | *-38.187* |  | *5.679* | *11.974* | *-1.265* | *17.850* |
|  |  | *(0.205)* | *(0.215)* | *(0.763)* | *(0.649)* |  | *(0.035)* | *(0.049)* | *(0.916)* | *(0.411)* |
| R squared |  | 0.1364 | 0.1376 | 0.1364 | 0.1389 |  | 0.1385 | 0.1458 | 0.1391 | 0.1460 |
| N |  | 145 | 145 | 145 | 145 |  | 144 | 144 | 144 | 144 |

**Note:** OLS regressions, dependent variable is *risk* [1,6]. *2D:4D^2^* is the square of *2D:4D* for quadratic models and *2D:4D X female* is the interaction variable for 2D:4D and female. Models 1-4 include analyses for the right hand, where models 5-8 for the left. *p-values* are given in parentheses.

**Table A2:** Regression Analysis of 2D:4D and sex specific risk predictions

| Model | (1) | (2) | (3) | (4) | (5) | (6) | (7) | (8) |
| --- | --- | --- | --- | --- | --- | --- | --- | --- |
| Dependent variable | *Predictions*  *about men* | *Predictions*  *about men* | *Predictions*  *about men* | *Predictions*  *about men* | *Predictions*  *about women* | *Predictions*  *about women* | *Predictions*  *about women* | *Predictions*  *about women* |
| *risk* | *0.229* | *0.232* | *0.224* | *0.226* | *0.175* | *0.172* | *0.177* | *0.173* |
|  | *(0.006)* | *(0.004)* | *(0.004)* | *(0.003)* | *(0.001)* | *(0.001)* | *(0.001)* | *(0.001)* |
| *2D:4D* | *-4.464* | *0.866* | *385.796* | *357.685* | *0.641* | *-4.503* | *-105.161* | *-57.490* |
|  | *(0.275)* | *(0.830)* | *(0.004)* | *(0.010)* | *(0.812)* | *(0.153)* | *(0.348)* | *(0.608)* |
| *2D:4D^2^* | *--* | *--* | *-201.280* | *-185.331* | *--* | *--* | *54.568* | *27.522* |
|  | *--* | *--* | *(0.004)* | *(0.010)* | *--* | *--* | *(0.347)* | *(0.634)* |
| *female* | *0.732* | *10.282* | *0.723* | *5.762* | *-0.064* | *-9.278* | *-0.061* | *-8.607* |
|  | *(0.002)* | *(0.154)* | *(0.002)* | *(0.412)* | *(0.681)* | *(0.069)* | *(0.693)* | *(0.079)* |
| *2D:4DXfemale* | *--* | *-9.906* | *--* | *-5.227* | *--* | *9.558* | *--* | *8.863* |
|  | *--* | *(0.186)* | *--* | *(0.473)* | *--* | *(0.071)* | *--* | *(0.081)* |
| *constant* | *7.115* | *1.998* | *-181.835* | *-169.563* | *0.578* | *5.515* | *51.803* | *30.992* |
|  | *(0.080)* | *(0.610)* | *(0.005)* | *(0.011)* | *(0.577)* | *(0.073)* | *(0.339)* | *(0.568)* |
| R squared | 0.1005 | 0.1117 | 0.1389 | 0.1418 | 0.1000 | 0.1198 | 0.1053 | 0.1211 |
| N | 145 | 145 | 145 | 145 | 145 | 145 | 145 | 145 |

**Note:** OLS regressions, dependent variables are *predictions_men* [1,6] in the first four models and *preditions_women* in the latter four. *2D:4D^2^* is the square of 2D:4D for quadratic models and *2D:4DXfemale* is the interaction variable for 2D:4D and female. p-values are given in parentheses.

**Table A3:** Regression analysis of left hand 2D:4D, risk predictions and gender bias in predictions

| Model | (1) | (2) | (3) | (4) | (5) | (6) | (7) | (8) |
| --- | --- | --- | --- | --- | --- | --- | --- | --- |
| *Dependent variable* | *predictions* | *predictions* | *predictions* | *predictions* | *gender bias* | *gender bias* | *gender bias* | *gender bias* |
| *Risk* | *0.185* | *0.189* | *0.187* | *0.188* | *0.048* | *0.046* | *0.044* | *0.049* |
|  | *(0.005)* | *(0.005)* | *(0.004)* | *(0.005)* | *(0.623)* | *(0.635)* | *(0.642)* | *(0.605)* |
| *Left 2D:4D* | *1.171* | *4.313* | *-42.563* | *-39.113* | *-6.718* | *-7.593* | *68.542* | *89.692* |
|  | *(0.659)* | *(0.319)* | *(0.045)* | *(0.288)* | *(0.105)* | *(0.098)* | *(0.087)* | *(0.188)* |
| *Left 2D:4D^2^* | *--* | *--* | *23.998* | *22.509* | *--* | *--* | *-41.298* | *-50.426* |
|  | *--* | *--* | *(0.058)* | *(0.230)* | *--* | *--* | *(0.081)* | *(0.153)* |
| *Female* | *-0.127* | *4.003* | *-0.170* | *0.801* | *0.749* | *-0.401* | *0.822* | *6.773* |
|  | *(0.530)* | *(0.429)* | *(0.409)* | *(0.904)* | *(0.009)* | *(0.952)* | *(0.003)* | *(0.512)* |
| *Left 2D:4D X female* | *--* | *-4.288* | *--* | *-1.005* | *--* | *1.194* | *--* | *-6.161* |
|  | *--* | *(0.415)* | *--* | *(0.883)* | *--* | *(0.863)* | *--* | *(0.566)* |
| *Constant* | *0.505* | *-2.527* | *20.346* | *18.404* | *8.133* | *8.977* | *-26.010* | *-37.914* |
|  | *(0.843)* | *(0.547)* | *(0.021)* | *(0.317)* | *(0.047)* | *(0.049)* | *(0.119)* | *(0.251)* |
| R squared | 0.0846 | 0.0880 | 0.0952 | 0.0953 | 0.0693 | 0.0695 | 0.0879 | 0.0912 |
| N | 144 | 144 | 144 | 144 | 144 | 144 | 144 | 144 |

**Note:** OLS regressions, dependent variables are *predictions about others* [1,6] in the first four models and gender bias in predictions, i.e. the difference between prediction about men and women in the latter four. *Left 2D:4D^2^* is the square of *Left 2D:4D* for quadratic models and *Left 2D:4D X female* is the interaction variable for *Left* *2D:4D* and *female*. *p-values* are given in parentheses.
